# Cell Cycle-Dependent Recruitment of Polycomb Proteins to the *ASNS* Promoter Counteracts C/ebp-Mediated Transcriptional Activation in *Bombyx mori*

**DOI:** 10.1371/journal.pone.0052320

**Published:** 2013-01-28

**Authors:** Zhiqing Li, Daojun Cheng, Hiroaki Mon, Li Zhu, Jian Xu, Tsuneyuki Tatsuke, Jae Man Lee, Qingyou Xia, Takahiro Kusakabe

**Affiliations:** 1 Laboratory of Silkworm Science, Kyushu University Graduate School of Bioresource and Bioenvironmental Sciences, Fukuoka, Japan; 2 State Key Laboratory of Silkworm Genome Biology, Southwest University, Chongqing, China; Università degli Studi di Milano, Italy

## Abstract

Epigenetic modifiers and transcription factors contribute to developmentally programmed gene expression. Here, we establish a functional link between epigenetic regulation by Polycomb group (PcG) proteins and transcriptional regulation by C/ebp that orchestrates the correct expression of *Bombyx mori asparagine synthetase* (*BmASNS*), a gene involved in the biosynthesis of asparagine. We show that the *cis*-regulatory elements of YY1-binding motifs and the CpG island present on the *BmASNS* promoter are required for the recruitment of PcG proteins and the subsequent deposition of the epigenetic repression mark H3K27me3. RNAi-mediated knockdown of PcG genes leads to derepression of the *BmASNS* gene via the recruitment of activators, including BmC/ebp, to the promoter. Intriguingly, we find that PcG proteins and BmC/ebp can dynamically modulate the transcriptional output of the *BmASNS* target in a cell cycle-dependent manner. It will be essential to suppress *BmASNS* expression by PcG proteins at the G2/M phase of the cell cycle in the presence of BmC/ebp activator. Thus, our results provide a novel insight into the molecular mechanism underlying the recruitment and regulation of the PcG system at a discrete gene locus in *Bombyx mori*.

## Introduction

Polycomb group (PcG) proteins are conserved transcriptional repressors involved in regulating body formation during embryonic development [1], and have also been characterized as chromatin modifiers required for the epigenetic regulation of numerous cellular processes, including cell cycle control, tumorigenesis, X-inactivation, cell fate decisions and differentiation [2], [3], [4], [5], [6].

PcG proteins function via three key multiprotein complexes: Polycomb repressive complex 1 (PRC1), Polycomb repressive complex 2 (PRC2), and Pleiohomeotic repressive complex (PhoRC) [7], [8], [9]. The PRC1 complex, which includes Polycomb (Pc), Polyhomeotic (Ph), Sex combs extra (Sce), and Posterior sex combs (Psc) in *Drosophila* or their counterparts in mammals, is involved in recognizing the chromatin marked with tri-methylated histone H3 on lysine 27 (H3K27me3) [8]. The PRC2 core subunits, which consist of Enhancer of zeste (E(z)), Extra sex combs (Esc), and Suppressor of zeste 12 (Su(z)12), are responsible for catalyzing the tri-methylation of H3K27 to produce H3K27me3 [7]. Importantly, PRC2 complex-mediated establishment of H3K27me3 is orchestrated by the recognition of Pleiohomeotic (Pho, a DNA-binding protein in the PhoRC complex) on specific DNA sequences called Polycomb responsive elements (PREs) in target genes [9], and by subsequent recruitment of other PcG components to the PREs region. A genome-wide search in *Drosophila* has revealed a conserved target site for Pho with a 17 bp binding sequence containing a core “CCATTTT” motif [9], which is the same as the binding sequence for Yin and Yang 1 (YY1), the mammalian ortholog of Pho [10]. More recently, a potential PRE region containing YY1-binding sites between the *HOXD11* and *HOXD12* locus has been identified in human embryonic stem cells, and the YY1-binding sites have been reported to contribute to the repression of this locus, but are not required for it [11]. On the other hand, several studies have revealed that long non-coding (lnc) RNAs, short RNAs, or even CpG islands in target genes specifically regulate the recruitment of PcG complexes in mammals [12], [13], [14], [15]. Therefore, there is insufficient evidence that an YY1-binding sequence is required for the recruitment of PcG complexes in mammals.

We previously identified the conserved 13 PcG genes in the silkworm, *Bombyx mori* [16]. Interestingly, our microarray analysis revealed that the *asparagine synthetase* (*ASNS*) gene is similarly up-regulated after RNA interference (RNAi)-meditated knockdown of some PcG genes, such as *BmSCE*, *BmESC*, *BmPHO*, and *BmSCM* (encoding a protein that can interact with BmPho and contribute to transcriptional repression), in silkworm cells [17]. The *ASNS* gene encodes an enzyme product that catalyzes the biosynthesis of asparagine using glutamine and aspartate as substrates, and is extensively expressed in mammalian cells [18]. It has been reported that under the condition of amino acid or glucose deprivation, human *ASNS* gene transcription is activated by the amino acid response (AAR) or endoplasmic reticulum stress response (ERSR) pathway, respectively, through two response elements, nutrient-sensing response elements (NSRE)-1 and −2 within the *ASNS* promoter region [19], [20]. In mouse hematopoietic stem cells, *ASNS* expression can be activated by a basic leucine zipper transcription factor, CCAAT/enhancer-binding protein (C/ebp) [21]. One isoform of C/ebp, C/ebpβ, has also been shown to bind to the NSRE sequence within the human *ASNS* promoter and activate *ASNS* expression in response to nutrient stress [19]. Much is known about the activation of the *ASNS* gene, but relatively little is known about how this gene is negatively regulated in normal cells in which the basal transcription is maintained. Recently, however, a microarray screening confirmed that *ASNS* transcription was induced in mouse cells by a deletion of either *BMI1* or *SU(Z)12*, which are members of the PcG protein family in mammals [21]. This finding, together with our microarray data, implies that *ASNS* gene may be a conserved target gene for PcG-mediated epigenetic repression. However, the molecular mechanism underlying this potential regulation remains largely unknown.

In addition, the emerging evidence has also shown that most cancer cells display high levels of *ASNS* expression, suggesting that the ASNS protein has an important function in cancer progression [22]. Although the relationship between cancer progression and *ASNS* expression is not yet well defined, it would be reasonable to suppose that *ASNS* expression is precisely regulated so as to prevent cancer development in normal mammalian cells, especially by a potential suppression of PcG regulation.

To define the role of PcG proteins in the regulation of *ASNS* gene expression, we here analyzed the structure and function of the *ASNS* promoter in the context of the *ASNS* regulation in *Bombyx*. Our results demonstrated that the *Bombyx ASNS* (*BmASNS*) gene is regulated by PcG-mediated repression and BmC/ebp-mediated activation in a cell cycle-dependent manner. *BmPHO* and *BmSCM* RNA interference (RNAi) had little effect on *BmASNS* expression at the late G1 phase, suggesting that the dissociation of PcG proteins from chromatin would occur before DNA replication. A CpG island on the *BmASNS* promoter, and, to a lesser degree, several YY1-binding motifs are considered to play a crucial role in the transcriptional repression by recruiting PcG complexes. These findings, for the first time, reveal an epigenetic and cell cycle-specific regulation of *ASNS* gene expression by a PcG system.

## Results

### *Bombyx* Asns is a functionally conserved ortholog of human ASNS

**Figure 1:**
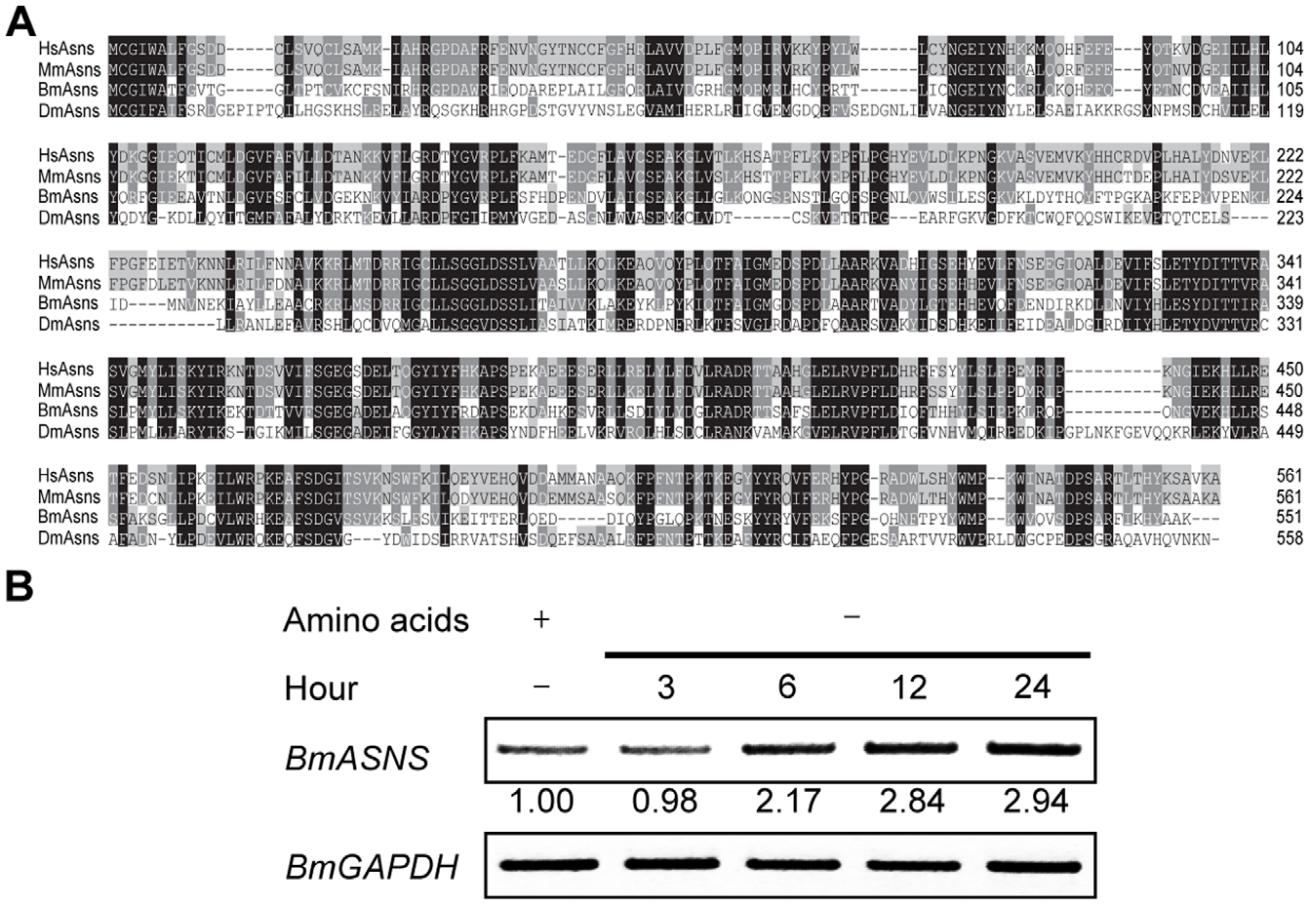
Asns is evolutionarily conserved in *Bombyx*. (A) Multiple alignments of amino acid sequences of Asns were performed by using ClustalX software. Identical and similar residues are highlighted in different shades. BmAsns, DmAsns, HsAsns, and MmAsns represented the Asns proteins from *B*. *mori*, *D*. *melanogaster*, *H*. *sapiens*, and *M*. *musculus*, respectively. (B) Expression of the *BmASNS* gene was elevated by amino acid deprivation. BmN4 cells were incubated in IPL-41 lacking amino acids. At the indicated time points, RNA was isolated and subjected to semi-quantitative PCR and the *BmGAPDH* gene was used as an internal control at each time point. The expression levels of the *BmASNS* gene were quantitated by ImageJ software and normalized to the *BmGAPDH* levels.

In order to understand the potential function and regulation of the *BmASNS* gene in *Bombyx*, we first cloned the full-length cDNA of the *BmASNS* gene according to the reported nucleotide sequence (NP_001037414.1). Amino acid sequence-based multiple alignment of Asns proteins from *Bombyx*, *Drosophila*, human, and mouse revealed high identities between insects and mammals (Figure 1A), raising the possibility that these Asns proteins share some similar functions.

In human, ASNS exhibits a functional response to nutrient stress [19]. Accordingly, we next examined whether *BmASNS* expression could also be induced under a deprivation of amino acid. To this end, we cultured silkworm cells using modified IPL-41 medium lacking all amino acid components and harvested at the indicated time points after the deprivation of amino acids for semi-quantitative PCR analysis. As shown in Figure 1B, the expression of *BmASNS* began to increase at 6 h after incubation with modified IPL-41 medium and peaked at 24 h. This result reveals that BmAsns is able to respond to amino acid limitation and may possess an enzymatic activity for amino acid biosynthesis. We also noted that the further loss of expression of the *BmASNS* gene mediated by RNAi under the condition of amino acid starvation significantly decreased the cell growth (data not shown), indicating an important role of BmAsns in cell proliferation. Taken together, these results led us to identify Asns as an evolutionarily conserved protein in *Bombyx* that can contribute to amino acid metabolism.

### *Bombyx* PcG proteins negatively regulate *BmASNS* expression

**Figure 2:**
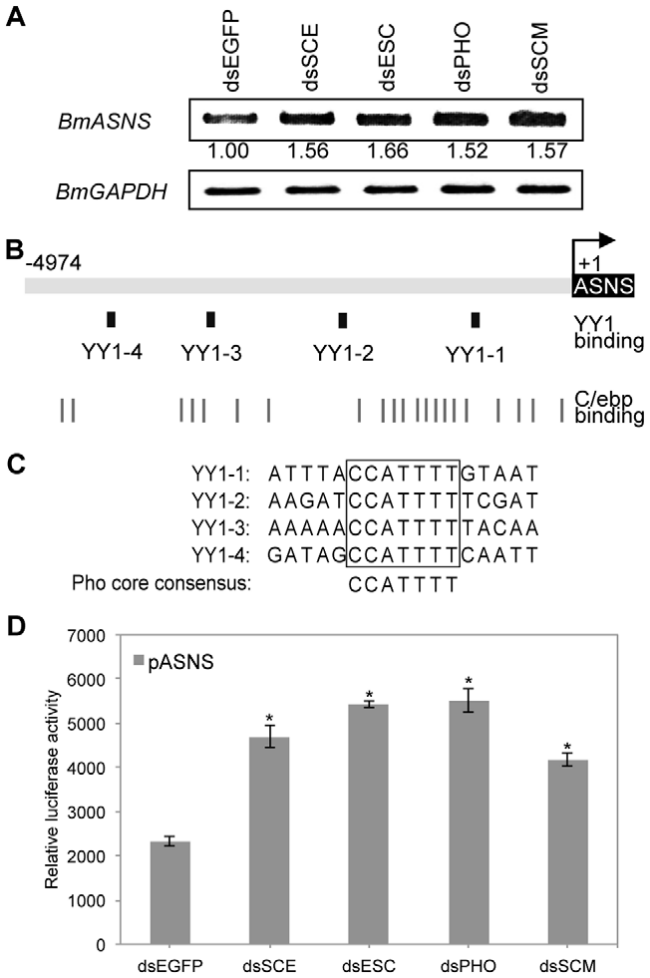
*Bombyx* PcG proteins negatively regulate *BmASNS* expression. (A) Knockdown of the silkworm PcG genes by dsRNA specific for *BmSCE*, *BmESC*, *BmPHO*, *BmSCM*, or *EGFP* (control)-mediated RNAi up-regulated the *BmASNS* gene expression, and the *BmGAPDH* gene was used as a loading control for normalization. (B) Schematic representation of the *BmASNS* upstream regulatory region containing numerous motifs for YY1 and C/ebp binding. (C) Sequence comparison of four putative YY1-binding motifs. The consensus sequence is boxed and consistent with the *Drosophila* Pho core-binding motif. (D) Activity of the transiently transfected *BmASNS* promoter was significantly increased by knockdown of the endogenous silkworm PcG genes mentioned in (A). The relative luciferase activity in each panel was calculated after normalization with the levels of transfected β-galactosidase expression. Data are shown as the mean ± SD of three independent experiments, *P<0.001, compared with the corresponding control.

Due to the potential role of BmAsns in regulating cell development and the abnormal expression of human ASNS in cancer cells, we sought to investigate the regulatory mechanism for this gene expression using the silkworm as a model. Our previous microarray analysis revealed that in silkworm cells, RNAi-mediated knockdown of PcG genes, such as *BmSCE*, *BmESC*, *BmPHO*, or *BmSCM*, all increased *BmASNS* expression [17]. Here, we also observed this up-regulation of *BmASNS* expression in PcG gene-silenced BmN4-SID1 cells by using a semi-quantitative PCR assay (Figure 2A).

To survey the effect of transcriptional regulation of PcG proteins on *BmASNS* expression, we isolated and cloned a 4,974 bp region upstream of the translational initiation codon of the *BmASNS* gene, as the promoter of *BmASNS* gene. Several prominent binding sites for YY1 protein were found in this region by the TFSEARCH program (http://www.cbrc.jp/research/db/TFSEARCH.html) (Figure 2B), and four conserved YY1-binding motifs in the region were shown to be identical to the Pho-binding profile in *Drosophila* (Figure 2C) [9]. Generally, YY1-binding sites mediate the regulation of YY1 protein as transcription factor on the expression of various genes including several PcG targets [23], thus we speculated that these YY1-binding sites may be similarly involved in the regulation of the *BmASNS* gene via the binding through BmPho, a *Bombyx* ortholog of the *Drosophila* Pho protein [16].

We also constructed a luciferase reporter system under the control of the *BmASNS* promoter and then introduced it into BmN4 cells to confirm this transcriptional regulation by measuring luciferase activity at 72 h after transfection. This promoter exhibited a stronger promoter activity compared with that of the promoter-less luciferase reporter plasmid (Figure S1). In agreement with the endogenous *BmASNS* expression in BmN4-SID1 cells (Figure 2A), knockdowns of each of four PcG genes, *BmSCE*, *BmESC*, *BmPHO*, or *BmSCM*, all increased the *BmASNS* promoter activity on the reporter plasmid (Figure 2D). Together, these results showed a negative regulation of PcG proteins on *BmASNS* expression and gave us a clue to elucidate the role of the PcG system in regulating the expression of a specific target gene in *Bombyx*.

### Transcription factor BmC/ebp activates *BmASNS* expression

**Figure 3:**
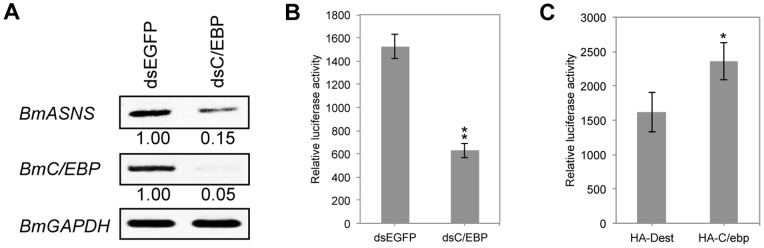
Transcription factor BmC/ebp activates *BmASNS* expression. (A) *BmASNS* expression was attenuated following the down-regulation of *BmC/EBP* by RNAi. (B, C) Activity of the transiently transfected *BmASNS* promoter was decreased or increased by knockdown (B) or overexpression (C) of the *BmC/EBP* gene, respectively. dsEGFP in (B) and HA-Dest (the backbone vector for constructing HA-C/ebp) in (C) were used as controls, respectively. The relative luciferase activity in each panel was calculated after normalization with the levels of transfected β-galactosidase expression. Data are shown as the mean ± SD of three independent experiments, *P<0.05, **P<0.001, compared with the corresponding control.

Transcription factor C/ebp has been demonstrated to activate *ASNS* expression in mammals [19]. Actually, we also found 21 putative C/ebp-binding sites on the *BmASNS* promoter (Figure 2B). Importantly, transcription of *BmASNS* was greatly decreased after the RNAi of the *BmC/EBP* gene in BmN4-SID1 cells (Figure 3A), which showed an important activation activity of BmC/ebp on *BmASNS* expression in the presence of the PcG proteins. In agreement with this endogenous promoter activity, a luciferase reporter assay revealed that the *BmASNS* promoter activity was decreased significantly after the silencing of *BmC/EBP* expression (Figure 3B) and increased after the BmC/ebp overexpression (Figure 3C). These findings thus indicated that transcription factor BmC/ebp is involved in activating *BmASNS* expression in *Bombyx*, as shown in mammals.

### YY1-binding sites on the *BmASNS* promoter are involved in the regulation of PcG proteins

**Figure 4:**
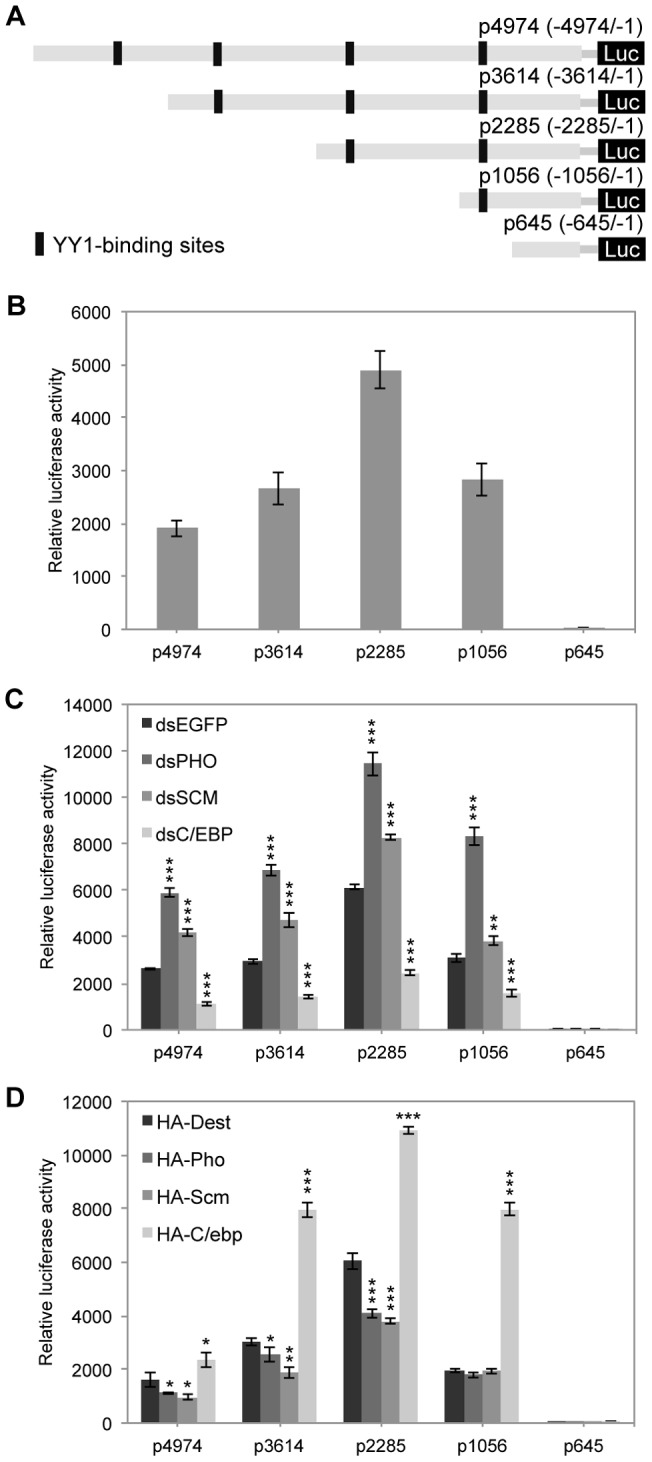
YY1-binding sites on the *BmASNS* promoter are involved in the regulation of PcG proteins. (A) Schematic representation of various *BmASNS* promoter truncates. The putative YY1-binding sites within this promoter were deleted serially. (B) Luciferase activities of different constructs transfected into BmN4 cells were measured after 72 h of transfection. (C) Knockdown of *BmPHO* or *BmSCM* induced the luciferase activities of different constructs, whereas down-regulation of *BmC/EBP* expression inhibited their activities. (D) Corresponding to the RNAi experiments, overexpression of BmPho, BmScm, and BmC/ebp inhibited or activated these truncated promoter activities, respectively. The relative luciferase activity in each panel was calculated after normalization with the levels of transfected β-galactosidase expression. Data are shown as the mean ± SD of three independent experiments, *P<0.05, **P<0.01, ***P<0.001, compared with the corresponding control.

To understand how PcG complexes repress *BmASNS* expression and how the different YY1 recognition sites on the *BmASNS* promoter are involved in this regulation, we performed a serial deletion analysis for the YY1-binding elements in the promoter (Figure 4A). Luciferase detection for each deletion construct of YY1-binding elements showed that the upstream region designated as p2285 with deletions of both YY1-3 and YY1-4 displayed the highest activity, suggesting that the activators are bound primarily between YY1-1 and YY1-2 (Figure 4B), the density binding for BmC/ebp activator in this region (Figure 2B), and the putative repressors will bind to the distal promoter region. In contrast, the p645 construct in which all four YY1-binding motifs were deleted showed very weak activity, indicating that the activator binding to the upstream sequence is necessary for basal transcription of the *BmASNS* gene. Together with the aforementioned findings, this result led us to speculate that PcG proteins would mainly bind to the YY1-3 and/or YY1-4 regions and repress *BmASNS* expression.

To examine this possibility, we performed RNAi experiments for four PcG genes, i.e., *BmPHO*, *BmSCM*, *BmSCE*, and *BmESC*, as well as the activator gene *BmC/EBP*. The RNAi of the PcG genes increased the activities of truncated constructs in p4974, p3614, p2285, and p1056, but not p645, whereas depletion of *BmC/EBP* decreased their activities (Figure 4C and Figure S2). Furthermore, we co-transfected these deletion constructs with overexpression plasmids for each of the PcG genes or BmC/ebp to test the effects of PcG genes or BmC/ebp overexpression on the activities of the *BmASNS* promoter. As shown in Figure 4D, the overexpression of BmC/ebp clearly up-regulated the promoter activities of all the constructs. This is consistent with the results obtained from the knockdown experiment, and may also suggest that its putative binding sites of the C/ebp protein are not occupied completely by the endogenous BmC/ebp and the ectopic expression of the BmC/ebp product will facilitate its own local recruitment to the unoccupied binding sites. On the other hand, the overexpression of BmPho or BmScm further repressed the promoter activity in p2285 and slightly repressed the promoter activity in p4974 and p3614. Combined with the RNAi experiments, these results suggested that the principal binding site present on p4974 and p3614 critical for the repression is pre-occupied by the PcG proteins.

### *Bombyx* PcG proteins catalyze a tri-methylation of H3K27 at the *BmASNS* promoter locus

**Figure 5:**
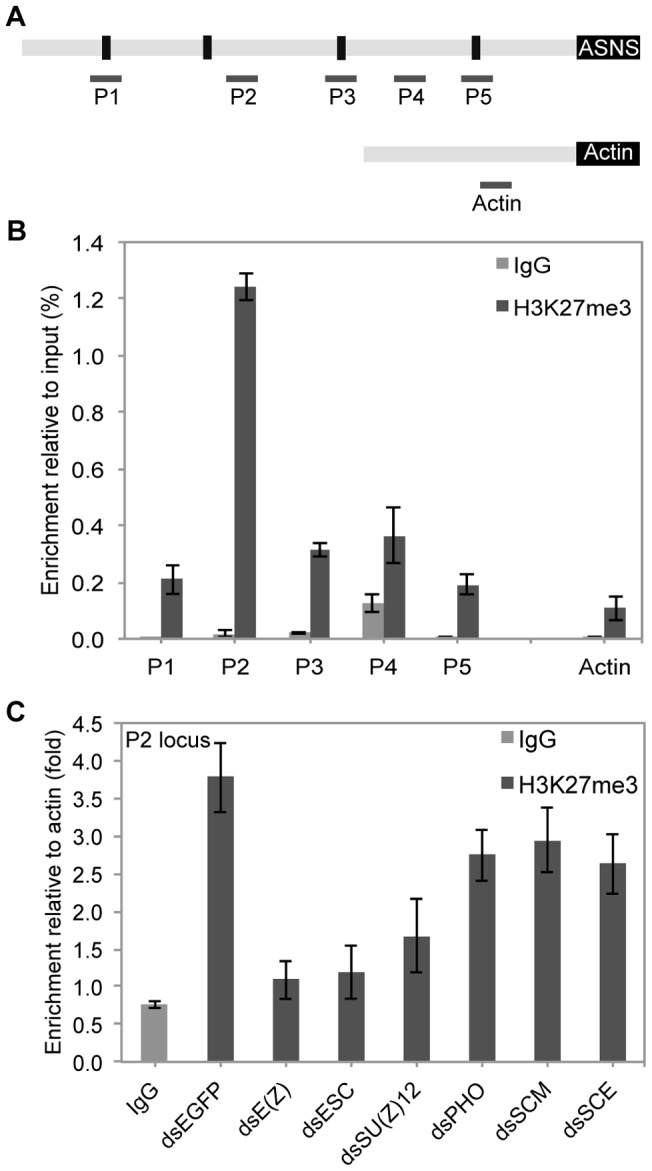
*Bombyx* PcG proteins catalyze a tri-methylation of H3K27 at the *BmASNS* promoter locus. (A) Schematic representation of the *BmASNS* promoter loci for ChIP assay. The primer sets were designed to span several YY1-binding sites and neighboring regions. The amplification for the promoter of the *BmACTIN* gene was used a negative control in the ChIP experiment. (B) The precipitation of DNA fragments from BmN4 cells by using a specific antibody for H3K27me3 was subjected to real-time PCR. The results showed that H3K27me3 was enriched in the *BmASNS* promoter, especially at the P2 locus. An antibody for IgG was used to show the nonspecific background signals. (C) BmN4-SID1 cells upon knockdown of different components of PcG complexes were collected for a ChIP experiment to detect the changes of H3K27me3 at the P2 locus. H3K27me3 enrichments at the P2 locus are presented as a ratio compared to the H3K27me3 signals at the control of the Actin locus in each column. Data are shown as the mean ± SD of three independent samples.

PcG complexes are responsible for the tri-methylation of H3K27 into H3K27me3 on its targets and this H3K27me3 mark is known to be required for PcG-mediated gene silencing [24]. To understand the mechanism by which PcG complexes suppress the *BmASNS* promoter activity, we analyzed the H3K27me3 patterns within the *BmASNS* promoter by ChIP assay with a specific antibody for H3K27me3. Pairs of primers for qPCR of the *BmASNS* promoter were designed as shown in Figure 5A. The results revealed that, although there was a broad domain of H3K27me3 enrichment across the *BmASNS* promoter locus, it was particularly enriched in the P2 region, namely between YY1-2 and YY1-3 (Figure 5B). This observation addressed why deletions of YY1-3 greatly increase the promoter activity (Figure 4B).

To determine whether the tri-methylation of H3K27 at the P2 region was mediated by the PcG system, a ChIP-qPCR assay in PcG gene-silenced BmN4-SID1 cells was performed. We found that the depletion of PRC2 components, such as *BmE(Z)*, *BmESC*, or *BmSU(Z)12*, greatly reduced the H3K27me3 marks (Figure 5C), which revealed that the PRC2 complex is required for the tri-methylation of H3K27 on the *BmASNS* promoter. In contrast, down-regulation of other genes, i.e., *BmPHO*, *BmSCM*, and *BmSCE* (one gene belongs to the PRC1 complex), did not induce a drastic reduction of H3K27me3 levels in comparison with the RNAi for PRC2 components. Therefore, it was shown that PcG complexes bind predominantly to the high-affinity binding site at the nearby P2 region of the chromosome, probably the YY1-3 site.

### The CpG island cooperates with YY1-binding sites and contributes to the repressive role of PcG proteins

**Figure 6:**
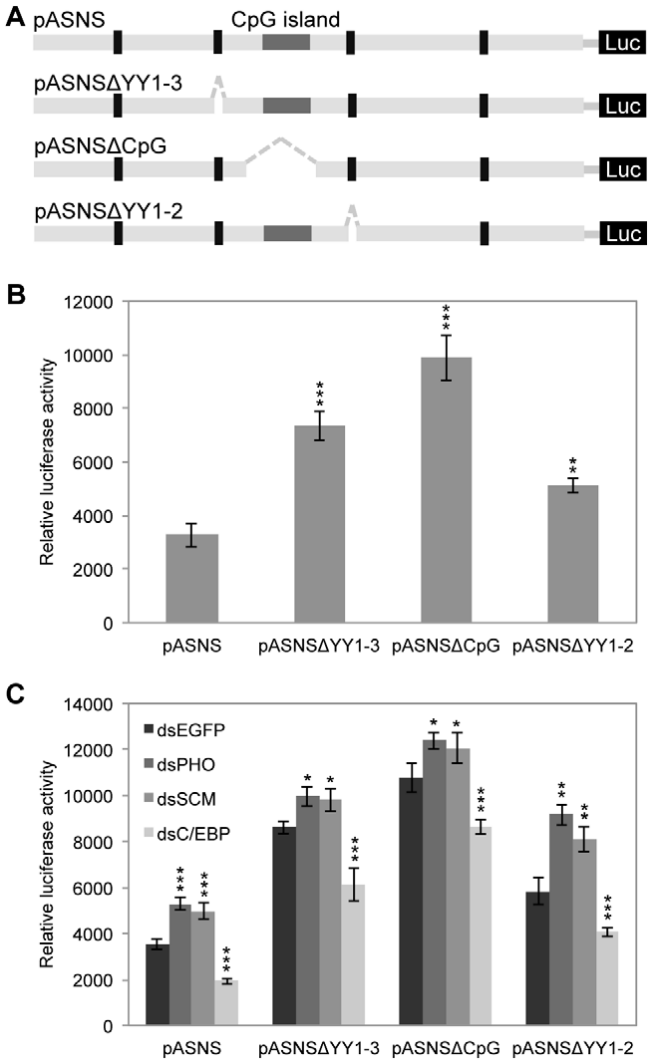
The CpG island cooperates with YY1-binding sites and contributes to the repressive role of PcG proteins. (A) Schematic representation of deletions of the YY1-binding site and the CpG island within the *BmASNS* promoter. A predicated CpG island is located between YY1-2 and YY1-3. (B) Deletion of the YY1-3 or the CpG island greatly up-regulated the *BmASNS* promoter activity than the deletion of YY1-2, and all of which were higher than the basic activity of pASNS construct. (C) Knockdown of *BmPHO* or *BmSCM* still promoted the activity of pASNSΔYY1-2 construct, but only with a modest increase in the activities of pASNSΔYY1-3 and pASNSΔCpG constructs. As expected, down-regulation of *BmC/EBP* reduced their activities. The relative luciferase activity in each panel was calculated after normalization with the levels of transfected β-galactosidase expression. Data are shown as the mean ± SD of three independent experiments, *P<0.05, **P<0.01, ***P<0.001, compared with the corresponding control.

In mammals, compared with the YY1-binding element, the CpG islands appear to play a more important role in recruiting of the PRC2 complex; and also, the CpG islands participate in the regulation of PcG complexes [14], [15]. However, there has been no report about this role of the CpG islands in insect species, maybe because insect genomes are poorly methylated [25]. Interestingly, we found a putative CpG island between YY1-2 and YY1-3 on the *BmASNS* promoter (Figure 6A). In order to check whether or how this CpG island is involved in the regulation of PcG complexes, we constructed the luciferase reporters in which YY1-2, YY1-3, or the CpG island was deleted from the *BmASNS* promoter construct, respectively (Figure 6A).

The luciferase promoter assay showed that removal of the CpG island significantly increased the luciferase activity compared to that of the YY1-3 deletion construct, and that the levels of both activities were higher than that by the deletion of YY1-2, which findings together suggested a critical repressive effect of the CpG island and YY1-3 on the *BmASNS* promoter activity (Figure 6B). *BmC/EBP* RNAi greatly decreased the promoter activity in each of these deletion constructs. This might be because there are only two C/ebp-binding sites between the region of YY1-3 and YY1-2 (Figure 2B) and the remaining C/ebp-binding sites could provide enough binding opportunity to BmC/ebp protein. Importantly, deletion of the YY1-2 site increased the promoter activity, but this construct still responded to RNAi for *BmPHO* or *BmSCM* (Figure 6B and 6C). In contrast, the activity in constructs resulting from deletion of the CpG island or YY1-3 exhibited a partial loss of the responses to *BmPHO* or *BmSCM* silencing. These findings indicated that the CpG island within the *BmASNS* promoter is involved in the PcG-mediated repression of *BmASNS* expression and may facilitate the binding of BmPho protein to the YY1-3 site near the H3K27me3-enriched P2 region, and the YY1-2 site may play only an auxiliary role in the repression of *BmASNS* expression.

### *Bombyx* PcG proteins counteract BmC/ebp activity to repress *BmASNS* expression

**Figure 7:**
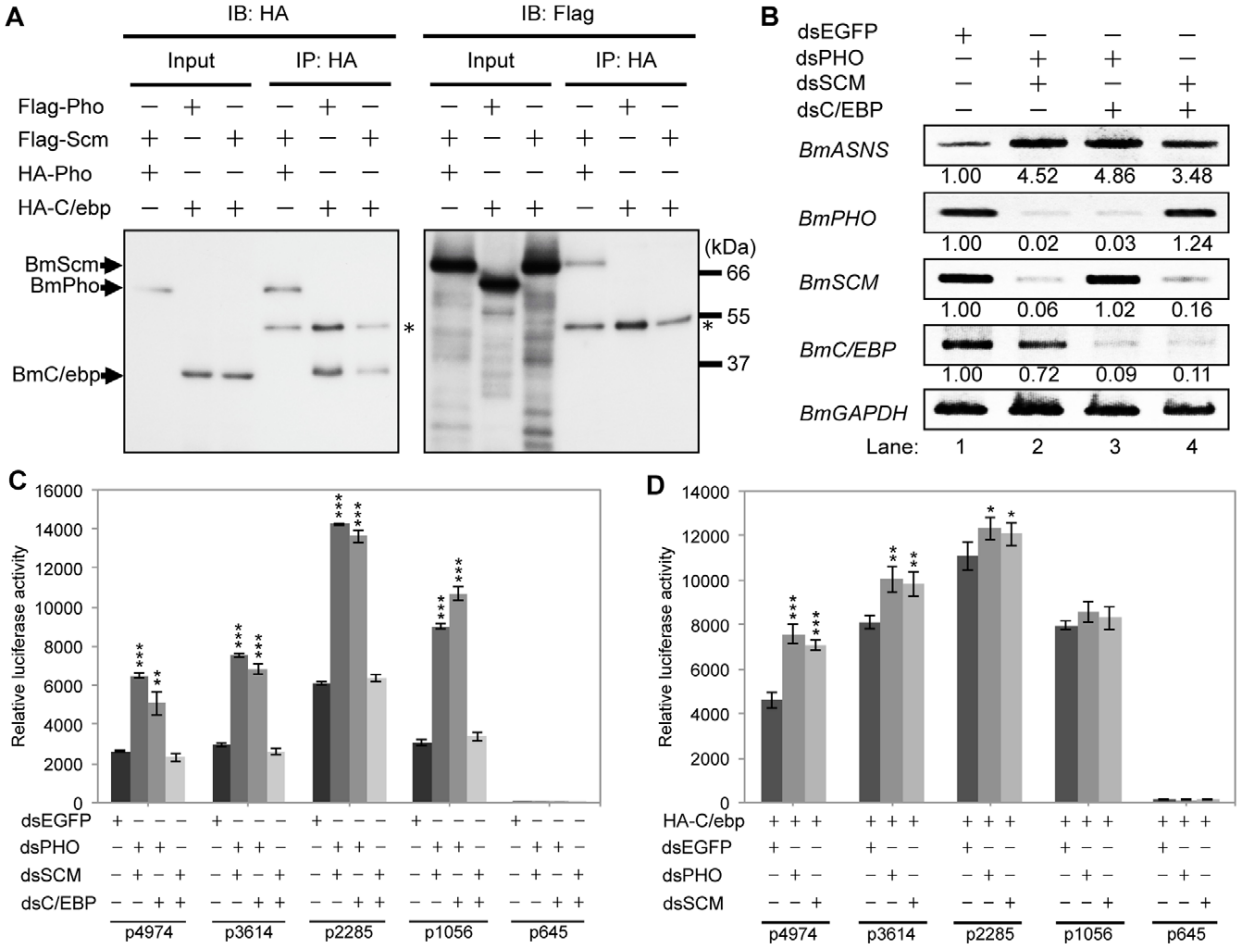
*Bombyx* PcG proteins counteract BmC/ebp activity to repress *BmASNS* expression. (A) Co-immunoprecipitation experiments were performed using repressors of BmPho, BmScm and the activator of BmC/ebp. Flag-tagged BmPho or BmScm was co-transfected with HA-tagged BmPho or BmC/ebp into BmN4 cells. Cell extracts were prepared in RIPA buffer in the presence of anti-HA antibody-coupled protein G beads. The resulting immunoprecipitates were applied to Western blotting analysis using the antibodies indicated in the graph. Asterisks represent the heavy chain of IgG. (B) Knockdowns of both activator and repressor up-regulated the *BmASNS* gene expression by semi-quantitative PCR analysis. (C) Consistent with the PCR data, the luciferase activities of different constructs upon the corresponding RNAi showed similar results. (D) Under the condition of overexpression of the BmC/ebp protein, further knockdown of *BmPHO* or *BmSCM* also greatly increased the activities of p4974, p3614, and p2285 but not p1056 or p645. The relative luciferase activity in each panel was calculated after normalization with the levels of transfected β-galactosidase expression. Data are shown as the mean ± SD of three independent experiments, *P<0.05, **P<0.01, ***P<0.001, compared with the corresponding control.

PcG proteins and BmC/ebp were demonstrated to act as repressors and to act as an activator, respectively, of *BmASNS* expression. We further examined whether these two regulators could interact with each other to act on the *BmASNS* promoter. First, we performed a co-immunoprecipitation experiment that confirmed an interaction between BmPho and BmScm (Figure 7A), as reported previously [17]. However, neither BmPho nor BmScm interacted with BmC/ebp (Figure 7A), suggesting that the repression and activation of the *BmASNS* promoter are accomplished independently by two complexes.

We then analyzed the effects of double knockdowns of these repressors and activator on *BmASNS* promoter activity. As expected, the depletions of both *BmPHO* and *BmSCM* increased the promoter activity (Figure 7B, Lane 2). Interestingly, the depletions of both *BmPHO* and *BmC/EBP* resulted in a higher *BmASNS* expression than the *BmPHO* and *BmSCM* depletions (Lane 3), while knockdowns of *BmSCM* and *BmC/EBP* (Lane 4) resulted in a slight reduction compared with *BmPHO* and *BmC/EBP* knockdowns, but still higher than the control treatment, and together these findings indicated the significant repressive effects of the PcG proteins on *BmASNS* promoter activity. It should also be noted that there was a reduced expression of *BmC/EBP* in response to the *BmPHO* and *BmSCM* depletions (Lane 2), although the mechanism of these effects was unknown. However, this weak decrease of *BmC/EBP* did not affect the up-regulation of *BmASNS* expression (Lane 2) because the higher expression of *BmASNS* was observed in the other two lanes (Lanes 3 and 4) when suffered from a significant loss of *BmC/EBP* expression. In contrast to the transcription from the chromosomal *BmASNS* locus, the effect of *BmPHO* knockdown on the deletion constructs was different from that of *BmSCM* RNAi in the absence of BmC/ebp (Figure 7C). These facts implied that the DNA-binding protein of BmPho has a dominant effect on the negative regulation of *BmASNS* expression; namely, BmPho alone is enough to exclude the putative transactivators other than BmC/ebp. Moreover, the formation of PcG complexes at the *BmASNS* locus via the BmPho recognition can antagonize the BmC/ebp-mediated activation, since *BmPHO* and *BmSCM* RNAi in the presence of BmC/ebp caused similar up-regulation of the promoter activity (Figure 2 and Figure 4).

To further estimate the relative power balance between these two transcriptional regulators, we performed RNAi assays for PcG genes under the enforced expression of the *BmC/EBP* gene. Overexpression of *BmC/EBP* up-regulated the *BmASNS* promoter activity on all the deletion constructs (see Figure 7D and 7C). Interestingly, the knockdown of *BmPHO* or *BmSCM* still significantly increased transcription of the p4974 and p3614 constructs under the condition of *BmC/EBP* overexpression, and slightly increased transcription of the p2285, but not of the p1056 construct. These results showed that the BmC/ebp transactivator can basically compete with PcG proteins in the presence of the CpG island region.

### *Bombyx* PcG proteins regulate *BmASNS* expression at the appropriate time during the cell cycle

The above results have demonstrated that the PcG system is involved in the repression of *BmASNS* expression, but there remains a question as to why PcG complexes are able to regulate a “housekeeping” gene that differs from other well-documented PcG targets such as the *Hox* genes in *Drosophila* as well as in mammals. The repression might not proceed in the canonical fashion of PcG regulation on the *BmASNS* gene.

**Figure 8:**
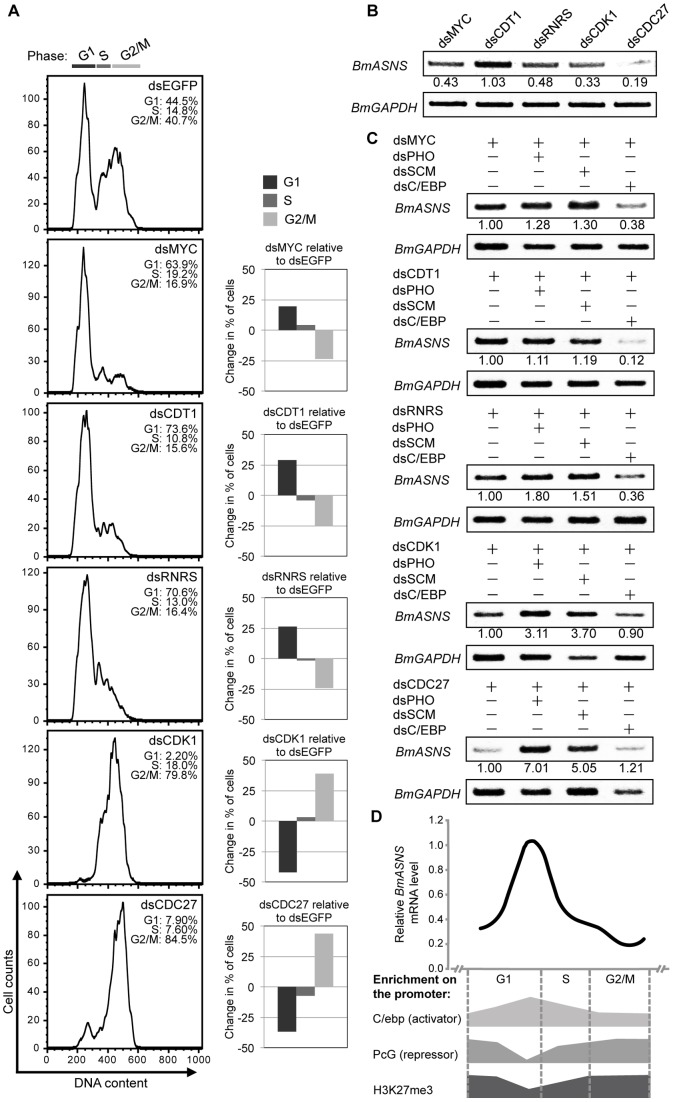
*Bombyx* PcG proteins repress *BmASNS* expression at the specific phases of the cell cycle. (A) A flow cytometer was used to measure the distribution of the cell cycle after knockdown of *BmMYC*, *BmCDT1*, *BmRNRS*, *BmCDK1*, *BmCDC27*, respectively, and the *EGFP* dsRNA treatment was shown as a control. Cells in G1, S, or G2/M were quantitated by FlowJo software. The right panels show the change in the percentage of cells in each phase of the cell cycle between the two indicated treatments. (B) Expression profile of the *BmASNS* gene during the cell cycle. The *BmASNS* gene was clearly down-regulated at the G2/M phase and up-regulated at the late-G1 phase. (C) Further knockdown of *BmPHO* or *BmSCM* in the cells arrested at the G1 phase had no or modest effects on *BmASNS* expression. By contrast, when cells were arrested at the G2/M phase, down-regulation of *BmPHO* or *BmSCM* greatly up-regulated *BmASNS* expression. In these phases, knockdown of *BmC/EBP* changed *BmASNS* expression, especially in G1- and S-arrested cells. (D) Model for the cell cycle-dependent recruitment of PcG complexes and C/ebp on the *BmASNS* promoter. The relative expression levels of the *BmASNS* gene were quantitated by ImageJ software and normalized to the *BmGAPDH* levels according to the expression profile of the *BmASNS* gene from (B). The representative enrichments of PcG complexes, H3K27me3, and C/ebp on the *BmASNS* promoter are diagrammed from the present data. See the text for details.

Following the clue that human cells can be induced to express the *ASNS* gene at the G1 phase or before DNA replication [26], we examined the expression profile of the *BmASNS* gene during the cell cycle of silkworm cells. Because there was no effective method to synchronize the cell progression in silkworm cells, we developed a novel strategy that took advantage of cell cycle-regulated factors with the ability to induce cell arrest at different phases upon RNAi treatment [27]. After checking the knockdown efficiency of each gene in the BmN4-SID1 cells by semi-quantitative PCR experiments (Figure S3), we further confirmed, using flow cytometry analysis, that the cell cycle distribution in control treatment was consistent with the previous pattern [27], [28], and that cells lacking the *BmMYC* gene primarily accumulated at the G1 phase of the cell cycle, and *BmCDT1* knockdown resulted in an arrest at the late-G1 phase, whereas *BmRNRS* RNAi could affect entry of cells into the G2 phase. We also observed that the loss of *BmCDK1* would arrest cells at the G2/M phase and the loss of *BmCDC27* led to an effective arrest at the metaphase (Figure 8A) [28]. Based on the arrest of these cells, a semi-quantitative PCR estimation revealed that, as shown in Figure 8B, the expression levels of *BmASNS* were very low at the M phase (*BmCDC27* knockdown), and after cell division, the expression was up-regulated, particularly at the late-G1 phase (*BmCDT1* knockdown), consistent with the profile of the *ASNS* gene in human cells [26]. These observations confirmed that the *BmASNS* expression level differs during different phases of the cell cycle, and indicated that the PcG system allows timely control of *BmASNS* expression to accommodate the needs of cells.

To test this possibility, we further performed separate knockdowns of *BmPHO*, *BmSCM*, or *BmC/EBP* in the knockdown cells from the foregoing experiment, respectively. As shown in Figure 8C, when cells were arrested at the G1 or S phase (*BmMYC*, *BmCDT1*, or *BmRNRS* RNAi), the concomitant silencing of *BmPHO* or *BmSCM* did not greatly change the expression of the *BmASNS* gene. By contrast, a significant increase of *BmASNS* levels were shown in the cells arrested at the G2/M phase (*BmCDK1* or *BmCDC27* RNAi) when there was an additional depletion of *BmPHO* or *BmSCM*. On the other hand, the loss of *BmC/EBP* expression only caused significant decrease of *BmASNS* transcription in the cells that underwent *BmMYC*, *BmCDT1*, or *BmRNRS* RNAi treatment, but not in those that underwent *BmCDK1* or *BmCDC27* RNAi treatment. Collectively, these results revealed that PcG proteins cooperate with BmC/ebp to regulate the *BmASNS* promoter activity in a cell cycle-dependent manner.

## Discussion

In this study, we report the first evidence that the PcG system directs gene regulation in a given locus in *Bombyx* and elucidate a new mechanism under the regulation of the PcG complexes on its target gene *BmASNS*, namely, PcG complexes can counteract with the C/ebp transactivator in the specific cell phase of the cell cycle through the *cis*-regulatory elements of YY1-binding motifs and the CpG island present on the *BmASNS* promoter.

*Drosophila* PREs containing YY1-binding motifs for Pho binding serve as a platform for the binding of the Pho protein and other PcG proteins that participate in the modification of histone and compaction of chromatin in the local region [29]. Moreover, the YY1-binding sites in the silkworm *BmASNS* promoter have also been shown to be involved in recruiting the PcG complexes. However, what is different from the model in *Drosophila* is that the CpG island in the *BmASNS* promoter could facilitate this repressive effect from PcG proteins through a potential mechanism of increasing the quantity of PcG proteins recruited or elevating the repressive H3K27me3 marks on the neighboring histone. This is in agreement with the well-known observation on the involvement of CpG islands in the regulation of PcG complexes in mammals [14], [15]. It remains to be determined whether such CpG island-mediated regulations are common in insects.

In *Drosophila* and mammals, the PcG system generally controls the expression of target genes by influencing the status of H3K27 methylation deposited by the PRC2 complex in their promoter regions. Our ChIP assays revealed a high level of H3K27me3 on the *BmASNS* promoter, especially near the CpG island. Moreover, the H3K27me3 was maximally reduced after down-regulating the expression of PRC2 components, revealing that it is the PRC2 complex rather than other complexes that contributes to the tri-methylation of H3K27 in *Bombyx*. Thus, it is reasonable to consider that the formation of a condensed chromatin structure through H3K27me3 mediated by the PRC2 complex will result in a high repressive effect of the PcG system on the prompter activity, just as in our previous observation of the depletion of *BmESC* [17], one component of the PRC2 complex. In addition, it is noted that H3K27me3 can be inherited as epigenetic memory at the chromosomal locus [30], and may provide a repressive signal for the subsequent restructuring of the PcG system mediated by the PRC1 complex [31]. However, because there is no epigenetic memory in the episomal construct newly introduced into the cell, our results demonstrated that BmPho or BmScm should play a more critical role in the creation of new epigenetic memory at the episomal construct, compared to the chromosomal locus. We know that this hypothesis is not sufficient to explain why the knockdown of each PcG gene induces similar effects on the chromosomal locus. In other words, this observation raises the possibility that the repression of *BmASNS* may be ascribed to the binding of PcG proteins *per se* or the established H3K27me3. We have previously suggested the presence of at least two types of regulatory complexes, namely, the whole PcG system and Pho/Scm complex [17]. Taken together with the current results, this finding makes it apparent that the *Bombyx* PcG system is not enough to suppress the *BmASNS* promoter activity without the continuous expression of the BmPho or BmScm protein, suggesting that the complex of BmPho/BmScm will be required to facilitate this repression from the intact PcG complexes. Indeed, we observed a similarly repressive effect of BmPho or BmScm overexpression on *BmASNS* promoter activity using the episomal constructs. Therefore, all these findings suggest that the removal of PcG proteins from this region is more crucial than the loss of histone modification to achieve the activation of *BmASNS* expression.

In addition to H3K27 methylation on the target promoter mediated by PcG complexes, some studies have also shown that several mammalian PcG proteins, such as EZH2 as a mammalian PRC2 component and CBX7 as one component of the PRC1 complex, could recruit DNA methyltransferases (DNMTs) on the promoter of target gene to repress its expression [32], [33]. Therefore, it would be of interest to examine whether the repression of *BmASNS* expression from PcG complexes could also be regulated by DNMTs, though it should be noted that the silkworm genome was shown to be poorly methylated in a previous study [34]. Our present data excluded this possibility because the knockdowns of *Bombyx DNMT1* and *DNMT2* (only two *DNMT* genes are found in the silkworm genome) could not induce the expression of *BmASNS* (Figure S4), indicating that the repression of *BmASNS* should be attributed only to H3K27 methylation from the PcG system rather than to DNA methylation from the DNMTs.

A recent study reported that the mouse *ASNS* promoter contained the di-methylated histone H3 lysine 4 (H3K4me2) and tri-methylated histone H3 lysine 9 (H3K9me3) [35]. H3K9me3 is associated with the formation of constitutive heterochromatin, which contributes to the repression of gene expression, whereas H3K4me2 at the promoter is related to the activation of gene transcription. Although there has been no report concerning an increase of H3K4me2 in response to amino acid or protein deprivation, contrarily, increased H3K9me3 level and elevated acetylation of histone H4 are observed [35], suggesting that the multiple histone modifications on the mouse *ASNS* promoter may be involved in the regulation of its own expression. To determine whether the H3K9me3 also contributes to the regulation of *BmASNS* expression, we performed RNAi against *Bombyx* H3K9me3-related genes, *BmHP1a*, *BmHP1b*, and *BmSU(VAR)3–9* [36]. The semi-quantitative PCR examination of *BmASNS* expression revealed no significant changes compared with the control RNAi treatment (Figure S5). Although this observation cannot directly rule out the possibility that the H3K9me3 modification also appeared on the *BmASNS* promoter, it points to a role of PcG complexes-mediated H3K27me3 as a key repressive modification for tightly modulating the transcription of the *Bombyx BmASNS* gene.

Genome-wide expression analysis in mouse hematopoietic stem cells has shown that PcG proteins and C/ebp can regulate a large set of genes in a positive or negative manner [21]. In this study, we identified that *Bombyx* PcG proteins negatively regulate the *BmASNS* expression by counteracting the transactivator BmC/ebp. This case of the *BmASNS* gene in *Bombyx* is in agreement with the negative correlation of PcG proteins and C/ebp on target gene expression in mouse [21], and therefore suggests a common model in which most target genes orchestrated by PcG complexes tend to be cooperatively regulated by the C/ebp protein. It would be worth further investigation to determine the target genes of BmC/ebp in a genome-wide scale and to analyze their correlations in conjunction with our microarray data from the knockdowns of PcG genes [17].

Importantly, we have demonstrated that PcG proteins play roles in the regulation of *BmASNS* promoter activity at the specific phase of the cell cycle and provide a model for the dynamic regulation of *BmASNS* expression involved by PcG repressors and BmC/ebp activator as shown in Figure 8D. According to this model, before cells enter into the S phase, uptake of various nutrient elements, including amino acids, is required. To increase the *BmASNS* transcription at the late G1 phase, the cells have to remodel the chromatins around the *BmASNS* promoter followed by the release of PcG complexes and the increase of accessibility of the promoter to transcriptional activators, including BmC/ebp. We speculate that a small number of H3K27me3 marks will be maintained in the promoter at this cell phase, since the previous studies have revealed that tri-methylation of H3K27 can be maintained at sites of DNA replication and even during the cell division [37], and moreover, the remaining H3K27me3 can help the PRC1 complex recognize the locus on the *BmASNS* promoter in the next cell phase. After completion of the S phase, the expression of the *BmASNS* gene will return to a low level mediated by the rebinding of the PcG system and/or the Pho/Scm complex at the G2 and M phases. The presence of PcG complexes may in turn increase the levels of H3K27me3 and promote the compaction of local chromatins, whereas the Pho/Scm complex assists in the repression by the whole PcG complexes. The comprehensive impact of this procedure will lead, to a certain extent, to the loss of BmC/ebp binding and then attenuate the promoter activity. Notably, it will require a balance between the repressor and activator to maintain this expression state. This appears to be a reasonable model for the regulation of *BmASNS* by these two distinct complexes, although it remains to be determined what signal triggers the release of PcG complexes from this locus.

In conclusion, the present results have clarified a novel epigenetic regulation in which PcG complexes regulate *BmASNS* expression involving H3K27me3. Our data confirmed that PcG proteins suppress the transcription of the *BmASNS* gene by recruiting themselves to the putative YY1-binding motifs and the CpG island within the *BmASNS* promoter. It is therefore tempting to speculate that both the YY1-binding element and the CpG island are sufficient to recruit PcG complexes and subsequently deposit H3K27me3 to repress the target gene expression in *Bombyx*. In particular, this study provides important new insights into the mechanism underlying the dynamic regulation of PcG target gene by the PcG system during the cell cycle. Our data will also shed fresh light on the regulation of the *ASNS* gene in human, which could explain a potential mechanism for the tumorigenesis mediated by the PcG system via the ASNS functions.

## Materials and Methods

### Cell culture

The silkworm BmN4 cell line (a gift from Dr. Chisa Aoki, Kyushu University Graduate School) [38] and BmN4-SID1 transgenetic cell line [28] were maintained in our laboratory and cultured at 27°C in IPL-41 medium (Sigma) supplemented with 10% fetal bovine serum (FBS) (Gibco).

For the amino acid deprivation assay, the modified IPL-41 medium was prepared according to the manufacturer's protocol (Sigma) but without addition of all amino acid components.

### Multiple sequence alignments

The Asns protein sequences of *Bombyx mori* (NP_001037414.1), *Drosophila melanogaster* (NP_996132.1), *Homo sapiens* (AAA52756.1), and *Mus musculus* (AAA85125.1) were downloaded from NCBI (http://www.ncbi.nlm.nih.gov/). Sequence alignments were performed by using ClustalX software.

### RNA extraction and semi-quantitative PCR

Total cellular RNA was isolated and reversed according to the procedure described previously [16]. The expression profile of the *BmASNS* gene was evaluated by semi-quantitative polymerase chain reaction (semi-quantitative PCR) using gene-specific primers (Table S1), and the silkworm *glyceraldehyde-3-phosphate dehydrogenase* (*BmGAPDH*) gene was used as an endogenous control. The relative expression levels of the *BmASNS* gene were quantitated by ImageJ software and normalized to the *BmGAPDH* levels.

### Cloning of the silkworm *ASNS* gene

Based on the reported *BmASNS* gene sequence (NP_001037414.1), we designed primers to amplify and obtain the cDNA clone (Table S1). The cDNA product then was digested by *Xho*I (underlined in Table S1), cloned into a pENTR^TM^11 (Invitrogen) vector, and verified by DNA sequencing.

### Isolation of the silkworm *ASNS* promoter region

The upstream promoter region of *BmASNS* was obtained by PCR screening of a genomic DNA template extracted from the silkworm strain p50T using a set of primers (Table S1) to yield a 4,974 bp sequence (AB751507). Here, we designated the translation start site of the *BmASNS* gene as +1, and thus this fragment was −4974/-1. The resulting product was digested with *Apa*I (underlined in Table S1), and inserted into the *Apa*I-*Eco*47III site of pXINSECT-DEST38 (Invitrogen). The recombinant plasmid was termed pASNS-DEST38 and its nucleotide sequences were determined by DNA sequencing.

For the reporter assay, the reporter plasmid (pASNS-Luc) was made by recombinant reaction between pASNS-DEST38 and pENTR^TM^11-Luc according to the gateway reaction system. pENTR^TM^11-Luc vector was generated using a pENTR^TM^11 (Invitrogen) vector into which the Firefly luciferase reporter gene was cloned [39].

### Sequential deletion constructs

Based on the *BmASNS* upstream sequence, we further designed the specific primers (Table S1), and amplified the fragments −3614/-1 (p3614), −2285/-1 (p2285), −1056/-1 (p1056), and −645/-1 (p645). Each fragment was subcloned into the same pXINSECT-DEST38 vector to construct the corresponding report vector.

For the constructs with specific deletion of *BmASNS* promoter region, such as pASNSΔYY1-3-Luc, pASNSΔCpG-Luc, and pASNSΔYY1-2-Luc, the direct amplification was performed based on a pASNS-Luc plasmid using the primers in Table S1. Then the PCR product was directly ligated.

### RNA interference

Knockdown of genes was performed with the gene-specific double-stranded RNAs (dsRNAs) in BmN4-SID1 cells according to our previous procedure [16], and the dsRNA for the *enhanced green fluorescent protein* (*EGFP*) gene was used as a control in this study. The knockdown efficency for each RNAi gene was quantitated by ImageJ software and normalized to the *BmGAPDH* levels.

### Transient transfection and luciferase assay

For normal transfection, the BmN4 cells seeded in 24-well plates with a density of 0.5×10^5^ were transfected with the luciferase reporter vectors mentioned above or the control promoter-less vector pENTR^TM^11-Luc according to our previous method [17]. Cells were harvested 72 h after transfection for luciferase activity assay.

For RNAi transfection, the BmN4-SID1 cells were pre-cultured in 24-well plates with a density of 0.2×10^5^ and additional dsRNAs according to the requirements of experiment. After 72 h of incubation, the cells were transfected and analyzed with the same method described above.

The transfection efficiency between dishes was measured by cotransfection with the pEXP38-βgalΔIE-1 vector expressing a β-galactosidase, and the β-galactosidase activity was used to normalize luciferase activity data [17]. All experiments included at least three independent transfections and data are shown as the mean ± standard deviation (SD).

### Co-immunoprecipitation assay

Co-immunoprecipitation (Co-IP) was performed according to our previous procedure [17].

### Chromatin immunoprecipitation assay

Chromatin immunoprecipitation (ChIP) analysis was performed according to a modified protocol of NimbleGen Systems, Inc. (Roche). Briefly, BmN4 cells or BmN4-SID1 cells (treated with dsRNAs) were harvested by gentle centrifugation and resuspended in growth medium on ice for 10 min. Cells were cross-linked in a 1/10 volume of crosslinking solution and mixed gently by inversion. The reaction was stopped by the addition of a 1/20 volume of 2.5 M glycine solution. Cross-linked chromatin was sonicated for 30 min with cycles of 30 s on/30 s off at an M2 setting using a Biorupter sonicator (Diagenode) to produce chromatin fragments of 0.2∼0.5 kb on average. After centrifugation and quantification, 100 μg of the supernatants was used as an input and 1 mg of the supernatants was incubated with 2 μg of H3K27me3 antibody (Upstate, Cat. #17–622). A rabbit IgG (Upstate, Cat. #PP64B) was used as the nonspecific antibody control. The antibody-bound complex was precipitated by Dynabeads® Protein G (Invitrogen, Cat. no. 100.03D). The DNA fragments in the immunoprecipitated complex were captured using an MPC-S magnet and released by reversing the crosslinking at 65°C for 12 h, and then purified using a QIAquick PCR purification kit (Qiagen).

### Quantitative real-time PCR analysis

Purified DNA was amplified by quantitative real-time PCR (qPCR) using SYBR Green Master Mix (Applied Biosystems) on a Thermal Cycler Dice Real Time System (Takara). The primers used for the qPCR to amplify the *BmASNS* promoter sequence are shown in Table S1. All ChIP assays were performed in triplicate, and the averaged data are presented with standard errors.

### Flow cytometry assay

Cell cycle distributions were analyzed by measuring the cellular DNA content using a Flow cytometer (Millipore) according to the previously described procedure [28].

### Statistical analysis

The statistical significance of difference between the treated and the corresponding control was evaluated by the Student's t-test, and a P-value <0.05 was considered statistically significant.

## Supporting Information

Figure S1 **Activity of the constructed** ***BmASNS*** **promoter.** BmN4 cells were transfected with pASNS-Luc (*Luciferase* gene under the control of the *BmASNS* promoter) or pNone-Luc (*Luciferase* gene without the promoter) vector. The luciferase activities were measured 72 h after transfection and normalized to the levels of transfected β-galactosidase expression. *BmASNS* promoter activity was calculated as the fold of the luciferase activity of pASNS-Luc to that of pNone-Luc and pNone-Luc was set as 1.
(TIF)

Figure S2 **Knockdown of** ***BmSCE*** **or** ***BmESC*** **can also induce the luciferase activities of different constructs of the** ***BmASNS*** **promoter.** The RNAi treatment and luciferase measurements were performed as shown Figure 4. The relative luciferase activity in each panel was calculated after normalization with the levels of transfected β-galactosidase expression. Data are shown as the mean ± SD of three independent experiments, *P<0.001, compared with the corresponding control.
(TIF)

Figure S3 **Knockdown efficiency for cell cycle-regulated factors.** Knockdown of *BmMYC*, *BmCDT1*, *BmRNRS*, *BmCDK1*, or *BmCDC27* in the BmN4-SID1 cells specifically reduced the expression of the corresponding genes.
(TIF)

Figure S4 ***Bombyx*** **DNA methyltransferase genes are not involved in the regulation of** ***BmASNS*** **expression.** Knockdown of the *BmDNMT1* and *BmDNMT2* genes did not affect the expression level of *BmASNS* gene by semi-quantitative PCR analysis.
(TIF)

Figure S5 **H3K9me3 may play a minor role in the regulation of** ***BmASNS*** **expression in** ***Bombyx*** **.** Knockdown of H3K9me3-related genes, including *BmHP1a*, *BmHP1b*, and *BmSU(VAR)3–9* did not alter the expression level of the *BmASNS* gene by semi-quantitative PCR analysis.
(TIF)

Table S1 **List of primers used in this study.**
(TIF)
