# Supplementary figures and images for: Cell Cycle-Dependent Recruitment of Polycomb Proteins to the ASNS Promoter Counteracts C/ebp-Mediated Transcriptional Activation in Bombyx mori
Source: PLoS One. 2013 Jan 28;8(1):e52320. doi: 10.1371/journal.pone.0052320 (PMC3557315; doi:10.1371/journal.pone.0052320)

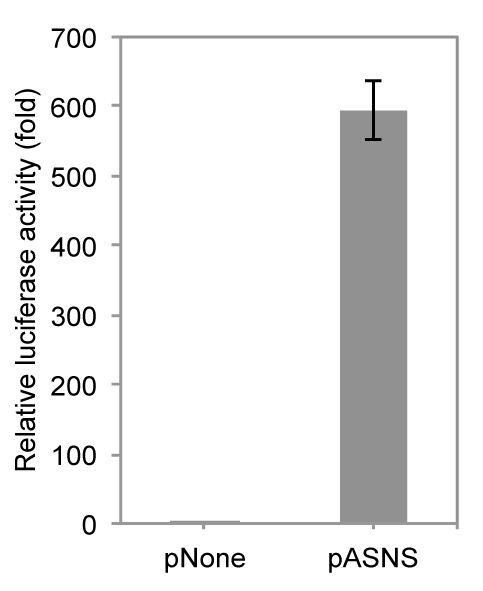

Supplement: Figure S1 — Activity of the constructed BmASNS promoter. BmN4 cells were transfected with pASNS-Luc (Luciferase gene under the control of the BmASNS promoter) or pNone-Luc (Luciferase gene without the promoter) vector. The luciferase activities were measured 72 h after transfection and normalized to the levels of transfected β-galactosidase expression. BmASNS promoter activity was calculated as the fold of the luciferase activity of pASNS-Luc to that of pNone-Luc and pNone-Luc was set as 1. (TIF) [file pone.0052320.s001.tif]

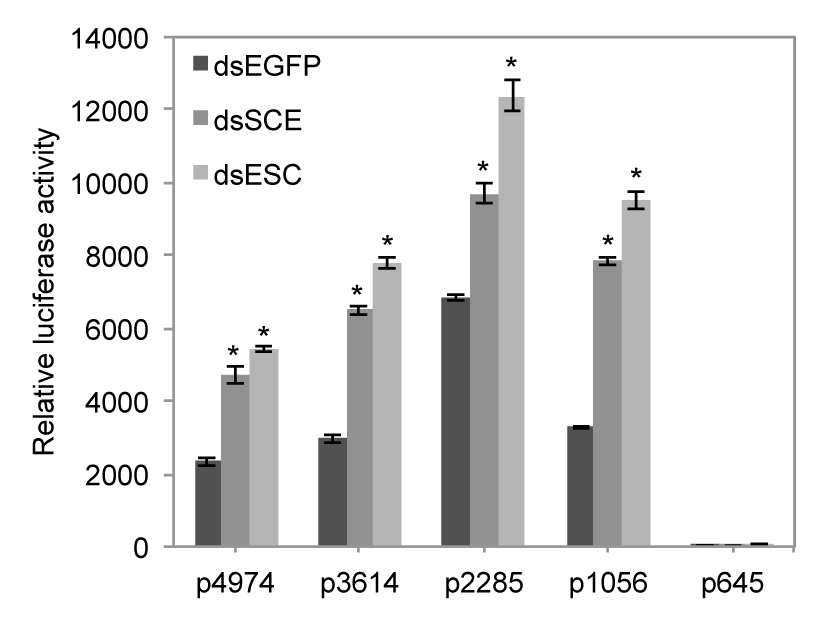

Supplement: Figure S2 — Knockdown of BmSCE or BmESC can also induce the luciferase activities of different constructs of the BmASNS promoter. The RNAi treatment and luciferase measurements were performed as shown Figure 4. The relative luciferase activity in each panel was calculated after normalization with the levels of transfected β-galactosidase expression. Data are shown as the mean ± SD of three independent experiments, *P<0.001, compared with the corresponding control. (TIF) [file pone.0052320.s002.tif]

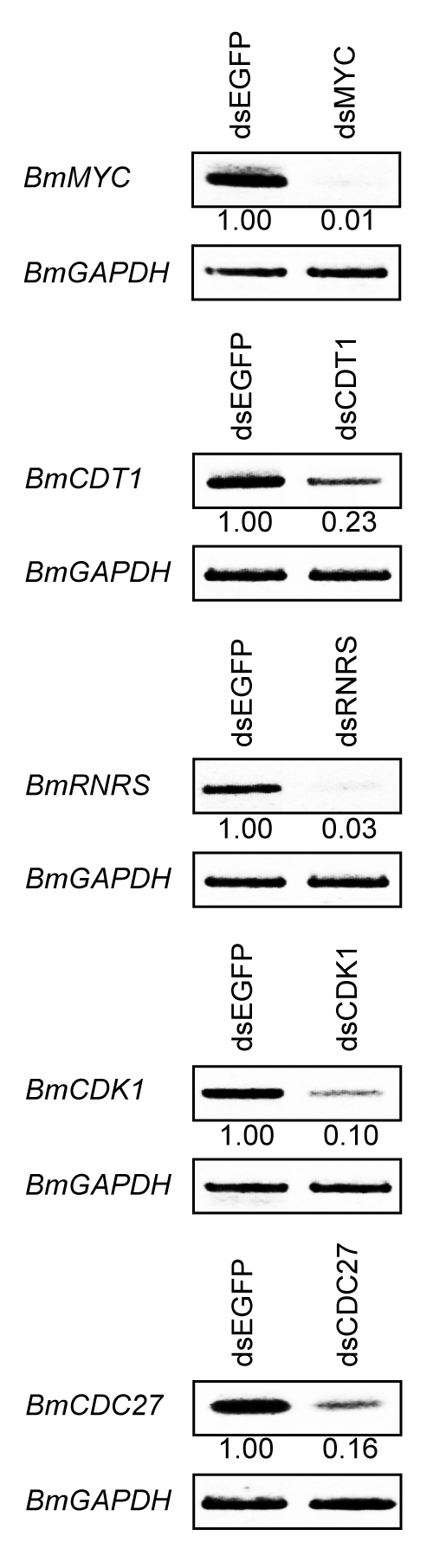

Supplement: Figure S3 — Knockdown efficiency for cell cycle-regulated factors. Knockdown of BmMYC, BmCDT1, BmRNRS, BmCDK1, or BmCDC27 in the BmN4-SID1 cells specifically reduced the expression of the corresponding genes. (TIF) [file pone.0052320.s003.tif]

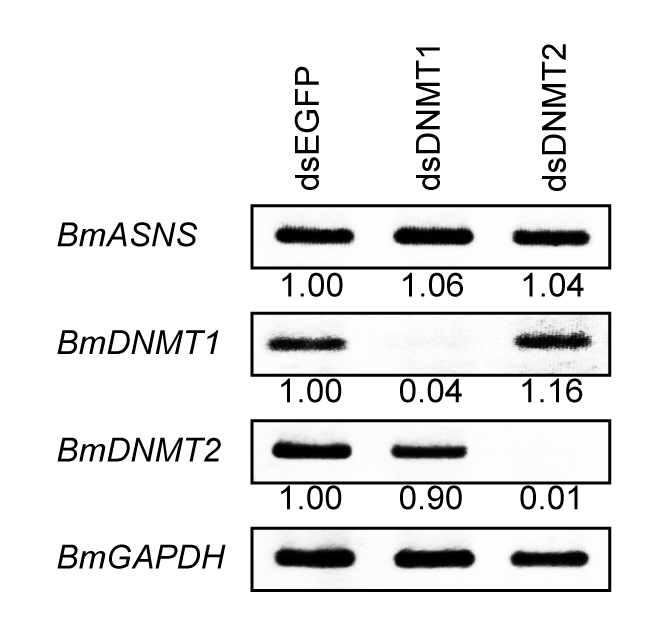

Supplement: Figure S4 — Bombyx DNA methyltransferase genes are not involved in the regulation of BmASNS expression. Knockdown of the BmDNMT1 and BmDNMT2 genes did not affect the expression level of BmASNS gene by semi-quantitative PCR analysis. (TIF) [file pone.0052320.s004.tif]

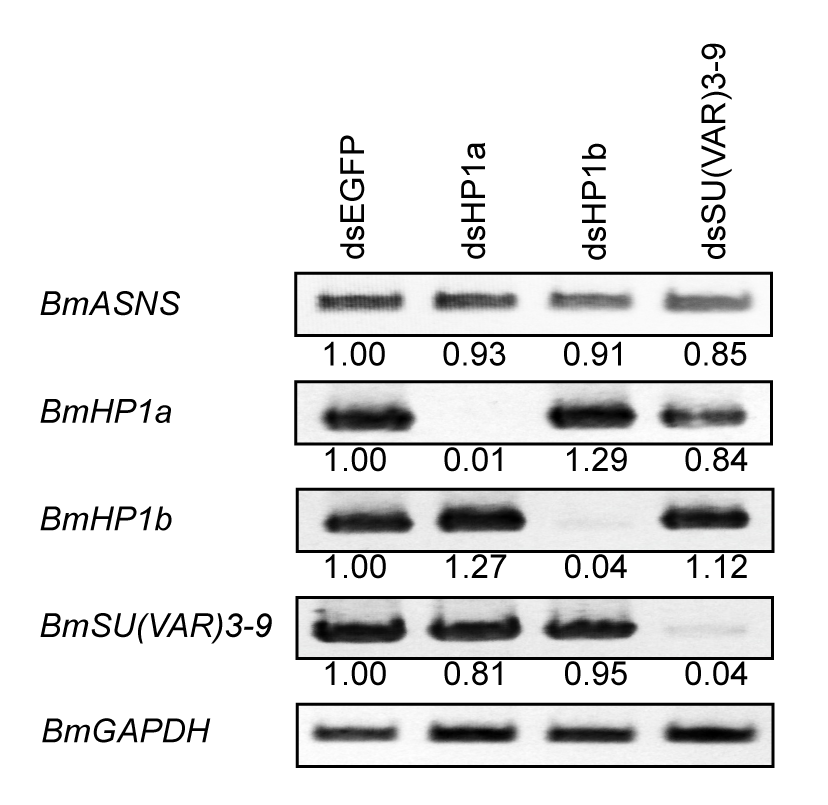

Supplement: Figure S5 — H3K9me3 may play a minor role in the regulation of BmASNS expression in Bombyx . Knockdown of H3K9me3-related genes, including BmHP1a, BmHP1b, and BmSU(VAR)3–9 did not alter the expression level of the BmASNS gene by semi-quantitative PCR analysis. (TIF) [file pone.0052320.s005.tif]

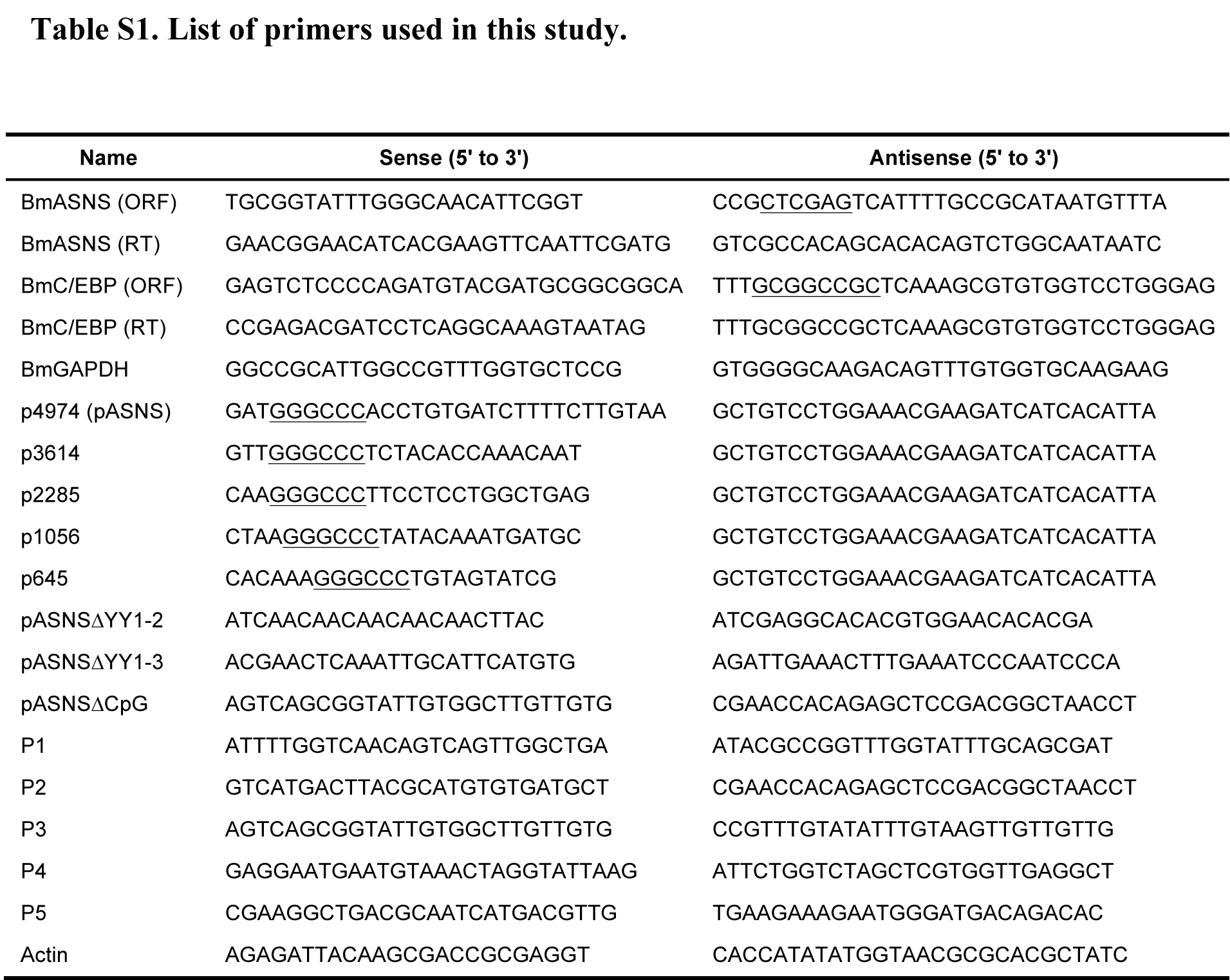

Supplement: Table S1 — List of primers used in this study. (TIF) [file pone.0052320.s006.tif]
